# A Review on Coexisting Giants: The Interplay Between Acromegaly and Diabetes Mellitus

**DOI:** 10.7759/cureus.64165

**Published:** 2024-07-09

**Authors:** Shailesh Aggarwal, Sweatha Mani, Abirami Balasubramanian, Keerthana Veluswami, Sudipta Rao

**Affiliations:** 1 Department of Internal Medicine, Jagadguru Sri Shivarathreeshwara Medical College, Mysore, IND; 2 Internal Medicine, K.A.P. Viswanatham Government Medical College, Tiruchirappalli, IND; 3 Internal Medicine, Stanley Medical College, Chennai, IND

**Keywords:** somatostatin analogues, pegvisomant, igf-1, diabetes mellitus, acromegaly

## Abstract

Acromegaly is a rare disease caused mainly by pituitary adenoma, which results in elevated growth hormone (GH) levels and its primary mediator, insulin-like growth factor (IGF-1). The condition causes various complications, including cardiovascular, respiratory, neuropsychiatric, metabolic, and gastrointestinal complications, which affect the patient's quality of life. Metabolically, there has been an increased incidence of acromegaly-associated diabetes mellitus (DM), IGF-1 being the primary mediator, affecting the patient's overall morbidity/mortality and associated surge in cardiovascular events. In the current state of medicine, both nonpharmacologic and pharmacologic approaches in managing acromegaly-associated DM are validated, having their own individualistic positive or negative impact on glucose metabolism. This review article has compiled studies to demonstrate a link between acromegaly. It summarises the existing data on acromegaly associated with DM, explicitly understanding the effect of various medical treatments on glucose homeostasis.

## Introduction and background

Acromegaly is defined as excessive production of growth hormone (GH) in adults, which originates from a monoclonal benign pituitary tumor (adenoma) in more than 90% of cases and is characterized by an acquired progressive somatic disfigurement (majority affecting the face and extremities) and associated systemic manifestation [1]. The term acromegaly is coined from the Greek terms "akros," meaning extremities, and "megas," meaning big, and was introduced by Pierre Marie, a renowned French neurologist at La Salpetrière Hospital in Paris. It was in 1886 when he first authored the initial account detailing the disease and its pathology. The prevalence of acromegaly ranges from 40 to 70 cases per million, and it exhibits an annual incidence of three to four new cases per million inhabitants, categorizing it as a rare disease [2]. However, a recent investigation in Belgium suggests that the prevalence of acromegaly might be higher than initially assumed, indicating an estimated prevalence ranging from 100 to 130 cases per million residents, signaling a potential underestimation of the occurrence of pituitary adenomas [3]. The typical age of onset for acromegaly is approximately 40 years, and it frequently goes undiagnosed until several years later, often ranging from four to more than 10 years after the initial onset, affecting both men and women equally [4-5].

Acromegaly stands out as a profoundly debilitating disease characterized by a range of severe comorbidities like cardiovascular, metabolic, respiratory, neoplastic, and musculoskeletal complications, collectively exerting a substantial impact on a patient's quality of life and elevating the risk of mortality [6-7]. Excessive GH levels impact insulin sensitivity, gluconeogenesis, and pancreatic β-cell function, contributing to glucose metabolism disruptions in many individuals with acromegaly [8]. GH exerts diabetes-inducing effects by increasing insulin resistance (IR) due to excessive fat breakdown (lipolysis) and abnormal fat distribution. This IR overshadows the insulin-sensitizing effects of IGF-1, predominantly due to more substantial metabolic effects of GH, resistance to IGF-1 or both [9]. The prevalence of DM among acromegalic patients is reported to vary between 20% and 56%, while the prevalence of glucose intolerance ranges from 16% to 46% [10]. Type 2 DM is associated with life-threatening complications, such as nephropathy, neuropathy, retinopathy, and macrovascular diseases, which result in excess risk of cardiovascular diseases and death.

The diagnosis of acromegaly is made through thorough clinical assessment and biochemical analysis. Clinically, the noticeable changes in physical appearance, including progressive enlargement of the extremities and changes in facial structure, are assessed through serial photographs. In biochemical analysis, through oral glucose tolerance test (OGTT), failure to suppress GH after administering oral glucose load makes the classical diagnosis of acromegaly. In addition, confirmation is obtained through an increase in the serum concentration of IGF-I, the primary GH-dependent growth factor, concerning the age-adjusted normal range. This combination of clinical and biochemical criteria is essential for a conclusive diagnosis of acromegaly [1]. The mainstay of acromegaly treatment involves the surgical removal of the GH-producing adenoma; however, nearly half of the patients necessitate additional therapy, including medical treatment and radiotherapy. The postoperative medical intervention typically involves using first-generation somatostatin analogs (SSAs), like octreotide or lanreotide, which not only diminish GH secretion and may shrink tumor remnants but also have the potential to decrease insulin secretion. This, in turn, may pose a risk of inducing DM in susceptible patients [11-12]. Second-generation SSAs such as pasireotide, GH-receptor antagonists like pegvisomant, and dopamine-2 receptor (D2R) antagonists like bromocriptine or cabergoline represent other categories of medical treatment. Scientific evidence suggests DM's prevalence and clinical significance in acromegaly, yet the underlying pathophysiology remains partially understood. In addition, the medications employed in treating acromegaly may directly affect insulin sensitivity or secretion. Moreover, more studies that explicitly address DM management in acromegaly need to be conducted.

In this article, we try to understand the effects of acromegaly on glucose homeostasis and the relationship between GH, IGF-1, and insulin signaling. Moreover, we aim to understand the impact of medications used to treat acromegaly and over-glucose homeostasis through available clinical and experimental data.

## Review

Pathophysiology

Acromegaly typically results from the presence of an anterior pituitary somatotrophic tumor that produces and releases excessive GH, also known as somatotropin. GH, a protein hormone, exerts its effects by binding to the growth hormone receptor (GHR) on the liver membrane and other target tissues [13]. In most instances, acromegaly is attributed to the presence of a pituitary adenoma that secretes GH. Other factors contributing to acromegaly include multiple endocrine neoplasia-1; McCune-Albright syndrome; ectopic pituitary adenoma located in the sphenoid region or parapharyngeal sinuses; iatrogenic elevation of GH levels; excessive release of the central growth-hormone-releasing hormone (GHRH) due to hamartoma, choristoma, or ganglioneuroma; and peripheral causes, such as small-cell lung cancer, medullary thyroid carcinoma, pheochromocytoma, adrenal adenoma, and insulinoma [14].

GH exerts physiological effects on glucose metabolism by causing peripheral IR in tissues through direct and indirect mechanisms. Directly, it induces processes like gluconeogenesis (synthesis of glucose from non-carbohydrate carbon substrate), glycogenolysis (breakdown of glycogen), and lipolysis, indirectly influencing insulin action through IGF-1 stimulation [15]. It hinders the insulin-induced suppression of hepatic gluconeogenesis and exerts lipolytic effects by generating free fatty acids (FFAs) from adipose tissue. This leads to competition between glucose and FFA as substrates and reduces glucose utilization in muscle tissues, thus elevating glucose production and plasma levels [16]. FFA delivery in the myocyte activates protein kinase C theta, which, in turn, favors serine over tyrosine phosphorylation of insulin receptor substrate 1 (IRS-1), resulting in diminishing phosphatidylinositol-3-kinase (PI3K) activity and subsequent insulin-stimulated glucose-transport [17,18]. An augmented insulin release from pancreatic β cells counters IR from elevated GH levels. However, as pancreatic secretory capacity diminishes over time, it weakens this compensatory mechanism, leading to the development of prediabetes and DM. Consequently, the decline in pancreatic β-cell function and reduced insulin secretion play a crucial role in the manifestation of glucose metabolism disorders [19]. Following the impairment of β-cell function, glucose metabolism disorders endure even after the resolution of acromegaly [20].

**Figure 1:**
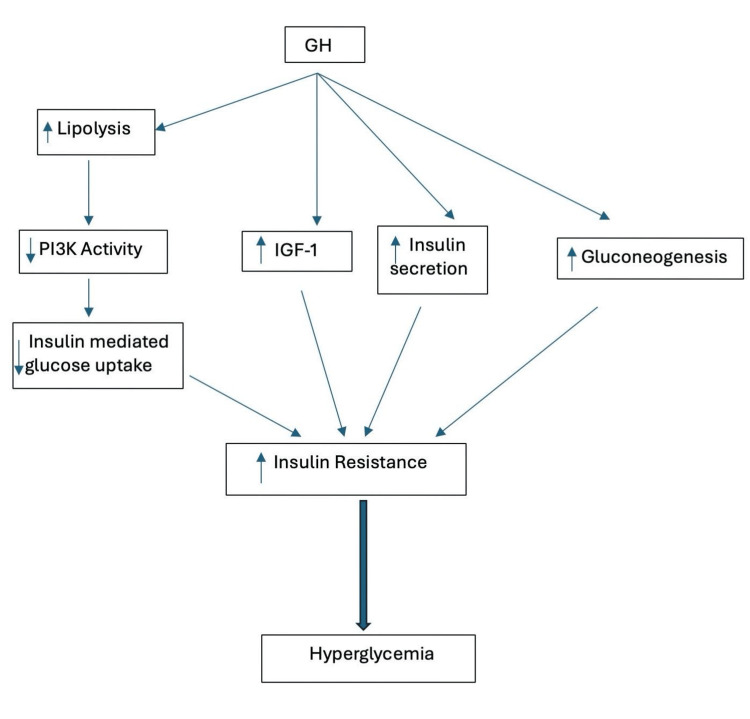
Pathophysiology between diabetes mellitus (DM) and acromegaly GH: growth hormone, IGF-1: insulin-like growth factor 1, PI3K: phosphatidylinositol-3-kinase Image credits: Shailesh Aggarwal

While IGF-1, the primary mediator of the effects of GH, usually contributes to maintaining glucose homeostasis, the heightened IR due to GH surpasses any potential positive impacts of IGF-1 on insulin sensitivity [8]. The coexistence of DM in individuals with acromegaly has been linked to elevated overall mortality rates, along with an increase in cardiovascular mortality and morbidity. In a Swedish observational cohort study, 254 patients diagnosed with both acromegaly and type 2 DM were compared with 532 individuals who had acromegaly without DM and various parameters of glucose metabolism like glycated hemoglobin (HbA1c), fasting plasma glucose (FPG), glucose levels after OGTT were compared over nine years. The study revealed that individuals with both acromegaly and concurrent type 2 DM experienced excess mortality, showing a 60% higher mortality rate compared to acromegalic patients without DM. Furthermore, mortality/morbidity attributed explicitly to cardiovascular causes was found to be doubled in those with acromegaly and DM, highlighting the significant impact of DM on cardiovascular health and overall mortality in individuals with acromegaly [21]. The mechanism is explained in Figure 1 [15,19].

Management

The management can be broadly divided into a nonpharmacologic group consisting of surgical and radiotherapy approaches and a pharmacologic group. The primary objectives in managing DM associated with acromegaly involve regulating levels of GH and IGF-I, minimizing tumor size, alleviating symptoms, addressing associated signs and comorbidities, and, ultimately, aiming to decrease mortality rates [22]. The primary choice for addressing acromegaly and related DM is surgical intervention, complemented by medical management to aid in preoperative tumor size reduction and complication prevention. In cases where surgery and medical approaches prove insufficient, radiation therapy has been employed as an adjuvant therapy for certain patients.

Nonpharmacologic (Surgery and Radiotherapy)

Transsphenoidal surgery stands as the primary and initial therapeutic choice in the management of acromegaly and associated DM. A Japanese study by Wasada et al. investigated six acromegalic patients with DM before and after adenomectomy. The researchers evaluated IR through the euglycemic hyper-insulinemic clamp technique and observed an improvement in insulin sensitivity following the surgical procedure [23]. The study's results can be compared with those of a separate investigation conducted by Battezzati et al. in Italy in 2003. The Italian study focused on 12 acromegalic patients with concomitant DM, examining their insulin sensitivity in glucose and protein metabolism before and after a successful adenomectomy. The outcomes of the study demonstrated a complete reversal of the previously observed alterations in glucose metabolism that were evident before the surgical intervention [24]. A study by Mori et al. aimed to determine the correlation between the reduction of IGF-1 levels and glucose tolerance and concluded a proportional relationship between improved glucose tolerance and the reduction of IGF-1 levels in the postoperative period [25]. In a similar study, Stelmachowska-Banas et al. [26] aimed to assess the reversal of impaired glucose tolerance and overt DM after transsphenoidal surgery and found that derangements in glucose levels and insulin sensitivity in acromegalic patients can be reversed after pituitary surgery. Kinoshita et al. evaluated 92 Japanese acromegaly patients who underwent successful pituitary surgery. IR, pancreatic beta cell function, GH, and IGF-1 levels were analyzed before and after the surgery, and the results showed that IR was primarily responsible for impaired glucose metabolism with the successful restoration of glucose levels after surgery, provided that the β-cell function was maintained [20]. However, if there is an impairment in β-cell function, abnormal glucose metabolism may persist over time despite the successful surgical cure of acromegaly [20].

Radiation therapy proves beneficial in cases where pituitary surgery has been unsuccessful, especially for acromegalic patients with extensive and infiltrating tumors or for those not effectively controlled by medical treatment. In a study conducted by Barrande et al., which involved 128 acromegalic patients, including 32 individuals with DM who underwent pituitary irradiation, there was observed improvement in blood glucose tolerance correlated with a subsequent reduction in GH levels [27]. Radiotherapy does not directly impact the management of DM in acromegaly patients; instead, it indirectly influences the management of DM by maintaining pituitary function and metabolic regulation. The primary aim of radiotherapy is to reduce or eliminate the pituitary tumor, thus helping to control symptoms and associated metabolic disturbances. A multidisciplinary approach and close monitoring are critical components in acromegalic patients to alleviate potential metabolic complications and ameliorate overall treatment outcomes.

Pharmacologic

Medical treatment consists of three classes of drugs: 1) GH (receptor antagonist, pegvisomant); 2) SSA (first generation (octreotide, lanreotide), second generation (pasireotide); 3. D2R analogs (bromocriptine and cabergoline).

Pegvisomant

Pegvisomant is a genetically engineered molecule designed to antagonize GH by directly interacting with the GHR [28]. A study by Rose et al. investigated the efficacy of pegvisomant in treating IR among five patients diagnosed with acromegaly. These patients were administered doses ranging from 15 to 30 mg/day of pegvisomant over a period ranging from 14 to 23 months and analyzed metabolic parameters, including IGF-1 levels, HbA1c, and FPG, which demonstrated that pegvisomant effectively improves IR in individuals with acromegaly irrespective of any weight loss experienced by the patients [29]. In contrast to first-generation SSAs, pegvisomant demonstrates superior effects on glucose homeostasis and insulin sensitivity [30]. Urbani et al. conducted a prospective study from December 2006 to 2010 involving 50 acromegalic patients with DM aimed to compare the effects of SSAs and pegvisomant on glucose control in these patients using parameters like OGTT, FPG, insulin concentrations, insulin sensitivity (QUICK-I), and homeostasis model assessment of insulin resistance (HOMA2-IR). They indicated that monotherapy using SSAs, pegvisomant, or combined therapy of SSA and pegvisomant improved IR. However, FPG concentrations and mean glucose concentrations were higher in SSA therapy alone when compared with pegvisomant therapy [31].

Similarly, using monotherapy or combined therapy, Jorgensen et al. analyzed the effects of SSAs and pegvisomant on glucose tolerance. They found the lowest plasma glucose levels during a two-hour OGTT when pegvisomant was used as monotherapy [32]. In a multicenter, open-label, 32-week trial study, Barkan et al. assessed the effects of switching octreotide-long acting release (LAR) therapy in 53 patients with pegvisomant (10 mg/day). They found improved fasting glucose levels and HbA1c after switching to pegvisomant [30]. For individuals who exhibit resistance to first-generation SSAs and struggle with uncontrolled DM in acromegalic patients, treatment with pegvisomant stands out as a favorable option [33].

SSAs

Octreotide and lanreotide are the first-generation SSAs and are considered the first line of medical therapy for treating acromegalic patients. They act by binding to high-affinity somatostatin receptor (SSTR) type 2 and SSTR type 5. They show weak affinity with SSTR type 3, which alters glucose metabolism by decreasing pancreatic insulin and glucagon secretion from the liver [34]. Cozzolino et al. conducted a meta-analysis of 47 interventional studies, including 1,297 acromegaly patients, and assessed the impact of SSAs over the entire glucose metabolic panel [35]. The research concluded that SSAs help exert their positive effects by lowering GH and IGF-1 levels but, in turn, result in worsening glucose metabolism by reducing insulin levels, thus increasing HbA1c and glucose levels after OGTT [35]. The former study shows similar results to a retrospective study conducted in Italy by Rhonchi et al., which evaluated 36 patients treated with six months of SSAs, advocating the need for strict monitoring of glucose homeostasis and lipid profile as SSAs were associated with a 1% increase in HbA1c levels and profound post-glucose insulin suppression [36]. James et al. evaluated 14 acromegalic patients receiving high-dose octreotide over 14 weeks and assessed insulin secretion response to standard 75g OGTT. They found reduced insulin secretion before and after the therapy [37].

While specific studies have suggested a negative impact on glycemic control among acromegaly patients treated with SSAs, contradictory findings have emerged from other research studies, which did not validate these outcomes. A meta-analysis study by Mazzioti et al. between 1987 and 2008 evaluated 31 studies with acromegaly patients treated with SSAs for at least three weeks and concluded that SSAs might lead to slight deterioration of glucose response to OGTT, with no significant impact over FPG and HbA1c levels [38]. In a prospective study, Baldelli et al. determined the effects of octreotide-LAR and lanreotide over glucose metabolism in 24 acromegalic patients. They found divergent results as both octreotide-LAR and lanreotide reduced IR but worsened the glucose levels at 120 minutes during OGTT due to impaired insulin secretion [39]. On the other hand, a post-hoc analysis by Mazzioti et al. investigated the effect of high-dose octreotide (60 mg/28 days) or high frequency (30 mg/21 days) on glucose homeostasis in 26 acromegalic patients. The study found no impact on glucose homeostasis in the majority of patients (16/26 patients), with worsening in (6/26 patients) due to uncontrolled acromegaly [40]. Similarly, a retrospective study by Couture et al. examined the glucose tolerance of 42 acromegalic patients primarily treated with lanreotide. The results indicated that the majority (60%) experienced no changes, 24% showed improvement, and 17% exhibited a decline in glucose tolerance associated with unsatisfactory control of GH levels [41].

Pasireotide, a second-generation SSA, appears to have a more pronounced negative impact on glucose levels compared to first-line SSAs, as evidenced by a randomized phase III study by Sheppard et al., which compared the effects of pasireotide-LAR with octreotide-LAR [42]. The study included 120 patients, of which 74 received pasireotide and 46 received octreotide. They were evaluated for up to 26 months and found that in terms of efficacy, pasireotide showed high efficacy with 48.6% of patients with biochemical improvement (GH <2.5 μg/L and normal IGF-1) compared to 45.7% in the octreotide arm [42]. However, hyperglycemia-related adverse events were observed in 62.9% of patients treated with pasireotide-LAR and 25.0% of patients treated with octreotide-LAR [42]. Insulin-producing beta cells express SSTR type 2 and SSTR type 5, whereas glucagon-producing alpha cells express SSTR type 2. Pasireotide binds with high affinity to SSTR type 5, decreasing insulin secretion. However, it negatively impacts glucagon secretion, which explains the insulin-glucagon imbalance leading to increased glucose levels [43]. In the pasireotide-LAR and pegvisomant (PAPE) study, an open-prospective study, 61 patients with acromegaly well controlled with SSA plus pegvisomant were switched with pasireotide-LAR monotherapy or in combination with pegvisomant. After 24 weeks of follow-up, IGF-1 levels were reduced to the reference range in 73.8% of patients, but the frequency of DM increased from 32.8% at baseline to 68.9%, explaining hyperglycemia as the most frequent adverse event [44].

The effects of SSA on glucose metabolism in acromegaly are summarized below (Table 1) [35-42,44]. In summary, the literature findings regarding the impact of SSAs on glucose metabolism in acromegaly suggest a slight deterioration in glucose levels attributed to the inhibition of insulin secretion. However, this effect appears clinically insignificant and is often counteracted by the reduction in GH levels, particularly in patients who effectively achieved disease control [28].

**Table 1:** Summary of somatostatin analogs OGTT: oral glucose tolerance test; HbA1c: glycated hemoglobin; BG: blood glucose; FPG: fasting plasma glucose; LAR: long-acting release; PAPE: pasireotide-long-acting release and pegvisomant

Authors	Study design	Drugs used	Number of studies/cases	Outcome measures	Conclusion
Cozzolino et al. (2018) [35]	Meta-analysis study	Lanreotide/octreotide	47 studies (1297 patients)	OGTT, HbA1c	Worsens glucose metabolism, reduces insulin levels, and increases HBA1c and glucose levels after OGTT
Rhonchi et al. (2006) [36]	Retrospective study	Lanreotide/octreotide	36 patients	BG, OGTT, HbA1c	Recommended glucose monitoring due to an increase in HBA1c levels
James et al. [37]	_	Octreotide	14 patients	Insulin secretion	Worsening of insulin secretion in response to OGTT
Mazzioti et al. (2009) [38]	Metanalysis study	Octreotide	31 studies	BG, OGTT, HbA1c	Minor changes in glucose levels after OGTT, but no significant impact on HBA1c and FPG levels
Baldelli et al. (2003) [39]	Prospective study	Octreotide/lanreotide	24 patients	OGTT, Insulin secretion	Divergent results with improved insulin sensitivity but impaired insulin secretion
Mazzioti et al. (2011) [40]	Post-hoc analysis	Octreotide	26 patients	OGTT	No significant impact over glucose homeostasis
Couture et al. (2012) [41]	Retrospective study	Lanreotide	42 patients	OGTT	No significant disturbances in glucose metabolism
Sheppard et al. (2014) [42]	Randomized control Phase III study	Pasireotide/octreotide	120 patients	BG	Pasireotide has more efficacy over octreotide for acromegaly treatment but more hyperglycemic adverse effects.
Muhammed et al. (PAPE study) [44]	Prospective study	Pasireotide	61 patients	HbA1c	The most frequent adverse event was hyperglycemia with increased frequency of DM in the pasireotide-LAR group.

Paltusotine, a novel SSA, has an agonistic action on SSTR type 2 [45]. ACROBAT Edge, a phase-II trial, evaluated the safety and efficacy of paltusotine in acromegalic patients who were treated with injections of SSAs. The primary endpoint was to observe the change in IGF-1 levels after 13 weeks and to observe a rise in IGF-1 levels after four weeks. No significant rise in IGF-1 levels was observed after 13 weeks (median IGF-1 change of -0.034). Of 22 patients, 18 patients showed a >20% rise in IGF-1 levels after the four-week washout period. Significant side effects observed were diarrhea (10.6%) and abdominal pain (8.5%) [46]. Currently, the drug is in a phase III trial, named PATHFINDER 1, to evaluate the safety and efficacy in acromegalic patients.

D2R analogs

Bromocriptine and cabergoline are D2R agonists in the pituitary gland and can suppress GH and prolactin secretion. D2R agonists affect glucose hemostasis by lowering central prolactin levels and increasing dopamine in the hypothalamic ventromedial and arcuate nuclei. This dopamine acts on pancreatic islets to stimulate insulin secretion and D2Rs on adipocytes, hepatocytes, and skeletal muscle to modulate their metabolic effects [47]. Bromocriptine therapy showed a beneficial effect on glucose metabolism, as demonstrated by a study by Rau et al. in which OGTT was performed in 12 acromegaly patients under treatment (15.0 +/- 6.8 mg/day for 12 +/- 3 years), which showed a reduction in basal and glucose-stimulated insulin levels in nine out of 12 patients, with GH reduction in all 12 patients [48]. Cabergoline has demonstrated superior efficacy in controlling acromegaly compared to bromocriptine [49]. There is a limitation of data regarding the effects of dopamine agonists on glucose metabolism in acromegaly patients. A prospective study by Higham et al. suggested that combination therapy with cabergoline and pegvisomant is more effective in reducing IGF-1 levels, with no significant changes in glucose metabolism after 18 weeks of treatment with either cabergoline (maximum dose 0.5 mg daily) or low-dose pegvisomant monotherapy [50].

In the current scenario, no specific guidelines have been implemented for managing DM in acromegalic patients; thus, a general approach using various antidiabetic drugs can be applied to acromegalic patients with deranged glucose metabolism, similarly used in non-acromegalic patients [28]. In a clinical trial of 70 patients, Cambuli et al. studied various treatments used for DM secondary to acromegaly. A percentage (15.7%) of patients achieved good glycemic control with diet alone, and 65.7% were managed with metformin, either used as monotherapy or combined with other hypoglycemic drugs, followed by insulin in 21.5%. The group achieved good glycemic control with a mean HbA1c of 6.4% [51]. The Acromegaly Consensus Group updated the consensus recommendations on diagnosis and treatment of acromegaly comorbidities using the Grading of Recommendations Assessment, Development, and Evaluation (GRADE) system and recommended monitoring fasting BG or OGTT every six months, particularly in patients with an uncontrolled disease or using SSAs and HbA1c every six months in newly diagnosed acromegalic patients with existing diabetes or prediabetes [52]. Metformin can be considered first-line therapy for DM and should follow the same guidelines as the general population [12]. Glucagon-like peptide 1-agonist and dipeptidyl peptidase inhibitors should be regarded as a second-line treatment on an individual basis [52].

Limitations

Acromegaly is a complex disorder associated with multiple comorbidities, such as hypertension, coronary artery disease, colorectal polyps, and cancer. This article focuses mainly on acromegaly associated with DM and does not discuss other comorbidities in detail. The effect of SSA analogs on glucose metabolism has yet to be determined, with studies using different glucose parameters still underway.

## Conclusions

According to the studies covered in this article, acromegaly is significantly associated with hyperglycemia and GH, IGF-1 being the primary mediators for the pathophysiology behind deranged glucose parameters in acromegalic patients. The severe IR caused by GH exceeds the counterregulatory mechanism of IGF-1 levels, which is one of the significant effects of glucose metabolism, and once the beta pancreatic cells die entirely out, permanent DM ensues, resulting in increased morbidity/mortality and increased chances of cardiovascular events. Management of DM associated with acromegaly is still a challenge due to the complexity of the condition and individual patient response to treatment. However, some modalities, such as surgery, radiotherapy, and pegvisomant, can improve glucose metabolism abnormalities. However, there is still a lack of literature regarding the universal approach and management of associated DM. An ideal treatment strategy should consider disease severity, comorbidities, medication tolerance, and patient preference. The primary goal of clinicians should be to achieve adequate GH levels by regularly monitoring blood glucose levels, insulin sensitivity, and other parameters and managing the associated DM with various antidiabetic drugs. We strongly believe in increased research efforts in acromegaly with associated DM to achieve a more goal-directed and patient-centered approach to improve patient outcomes.
